# Correction to European Consensus on Functional Bloating and Abdominal Distension—ESNM/UEG Recommendations for Clinical Management

**DOI:** 10.1002/ueg2.70183

**Published:** 2026-01-28

Melchior, C., Hammer H., Bor S., Barba E., Horvat I.B., Celebi A., Drug V., Dumitrascu D., Kalkan I.H., Hauser G., Lionis C., Livovsky D., Mari A., Mulak A., Surdea‐Blaga T., Tack J., Vanuytsel T., Savarino E.V., Zarate‐Lopez N., Hammer J., Dickman R. “European Consensus on Functional Bloating and Abdominal Distension—An ESNM/UEG Recommendations for Clinical Management.” *United European Gastroenterology Journal*: 2025;13:1613–1651. https://doi.org/10.1002/ueg2.70098.

On page 21 of the published article, “VSL#3, *Lactobacillus plantarum, Bifidobacterium infantis* 35,624, and *Bifidobacterium animalis* DN‐173 010 have also been effective in reducing bloating [249–252]” has been revised to “De Simone Formulation, *Lactobacillus plantarum, Bifidobacterium infantis* 35,624, and *Bifidobacterium animalis* DN‐173 010 have also been effective in reducing bloating [249–252].”

A number of studies cited in this paper (references 249–252) assessed a probiotic formulation previously known as VSL#3. The probiotic formulation that was assessed in these studies is now known by the generic name De Simone Formulation (DSF). The current product known as VSL#3 is not the same formulation as the De Simone Formulation or the pre‐2016 VSL#3. The De Simone Formulation is available under the brand names Visbiome (USA) and CDS22‐formula (Europe).

We apologize for the error.

